# Novel phthalide derivatives identified from *Ligusticum chuanxiong* (*Chuanxiong*)

**DOI:** 10.1186/s13020-016-0080-2

**Published:** 2016-03-08

**Authors:** Jun Yang, Xiao-Lin Feng, Yang Yu, Qi Wang, Jian Zou, Chuan-Xi Wang, Zhen-Qiang Mu, Xin-Sheng Yao, Hao Gao

**Affiliations:** Institute of Traditional Chinese Medicine and Natural Products, Jinan University, Guangzhou, China; Institute of Clinical Pharmacology, Guangzhou University of Chinese Medicine, Guangzhou, China

**Keywords:** *Ligusticum chuanxiong*, Chuanxiong, Phthalide fatty acid esters, New subtype of phthalides, Chuanxiongins

## Abstract

**Background:**

*Ligusticum chuanxiong* Hort. (*Chuanxiong*) is a well-known Chinese medicine, and studies on its chemical constituents are important for explaining its mechanism of action and quality control. This study aims to investigate the chemical constituents of the dried rhizome of. *L. chuanxiong.*

**Methods:**

The dried rhizome of *L. chuanxiong* was extracted with 60 % ethanol, and the concentrated extract was isolated by silica gel, octadecyl silane, and Sephadex LH-20 columns, followed by preparative/semipreparative high-performance liquid chromatography (HPLC) to obtain the pure chemical constituents. The structures of the constituents were elucidated by HR-ESI-MS, UV, IR, 1D NMR, and 2D NMR methods. Enantiomeric separation was achieved by a chiral HPLC method. The absolute configuration was determined by the modified Mosher’s method.

**Results:**

Six novel phthalide derivatives, (+)/(−)-chuanxiongins A–F (**1**–**6**), together with four known phthalides (**7**–**10**) were isolated from *Chuanxiong*. All of the new compounds (**1**–**6)** were present as pairs of enantiomers. Enantiomeric separation of **1** was successfully achieved by HPLC on a chiral column. The absolute configuration of (−)-**1** was determined by a modified Mosher’s method.

**Conclusion:**

The six novel phthalide derivatives (**1**–**6**) isolated from *Chuanxiong* were phthalide fatty acid esters that were structurally analogous and characterized by fatty acid acylation at 6-OH or 7-OH.

## Background

The dried rhizome of *Ligusticum chuanxiong* Hort. (*Chuanxiong*) has been used for the treatment of menstrual disorders in Chinese medicine [1]. *Chuanxiong* has the functions of activating blood flow, expelling wind, and relieving pain associated with *blood* (*xue*) and *qi* stagnation [2]. Previous phytochemical studies on *Chuanxiong* isolated some phthalides (monomeric and dimeric phthalides) [3–6], alkaloids [7], phenolic acids [8], ceramides, and cerebrosides [9]. Among them, phthalides as the characteristic constituents had bioactivities including vasodilatation [10], decreased platelet aggregation [11], analgesia [12], anti-inflammation [13, 14], and butyrylcholine esterase inhibition [15]. The chemical studies on the constituents of *Chuanxiong* and identification of new constituents are beneficial for explaining how it exerts its effects and form the foundation for its quality control. This study aims to investigate the chemical constituents of *Chuanxiong* and identify new constituents.

## Methods

### General experimental procedures

Optical rotations were obtained on a JASCO P-1020 digital polarimeter (JASCO Corporation, Tokyo, Japan). UV spectra were recorded with a JASCO V-550 UV/Vis spectrometer (JASCO Corporation, Tokyo, Japan). IR spectra were acquired using a JASCO FT/IR-480 plus spectrometer (JASCO Corporation, Tokyo, Japan). CD spectra were obtained on a JASCO J-810 spectropolarimeter at room temperature. 1D and 2D spectra were measured with a Bruker AV300/400 spectrometer in CDCl_3_ (*δ*_H_ = 7.26 ppm, *δ*_C_ = 77.2 ppm) or C_5_D_5_N (tetramethylsilane (TMS) as internal standard) solution. ESI-MS spectra were acquired on a FINNIGAN LCQ Advantage MAX mass spectrometer. HR-ESI-MS spectra were taken on a Waters Snapt G2 mass spectrometer. Analytical HPLC was performed on a Waters 2695 HPLC system equipped with a UV detector and a reversed-phase column (5 µm; i.d. 250 × 4.6 mm; Cosmosil). HPLC separations were performed using a Waters 1515 system equipped with a UV detector and a reversed-phase semipreparative column (5 μm; i.d. 250 × 10 mm; Cosmosil) or preparative column (5 μm; i.d. 250 × 20 mm; Cosmosil). Chiral separation was achieved by HPLC on a Dionex 3000 series pump equipped with a UV detector and a Lux TF Cellulose-2 column (3 µm; i.d. 250 × 4.6 mm; Phenomenex). Silica gel (200–300 mesh; Qingdao Marine Chemical Ltd., China), octadecyl silane (ODS) (YMC Ltd., Japan), and Sephadex LH-20 (Amersham Pharmacia Biotech, Sweden) were used for column chromatography. Mosher’s reagent was purchased from Alfa Aesar (England).

### Plant material

The dried rhizome of *L. chuanxiong* Hort. was supplied by Sichuan Neautus Traditional Chinese Medicine Co. Ltd. (China) in 2012, and identified by Yue-Cheng Li (Sichuan Institute for Food and Drug Control) by characteristic identification, microscopic identification, and the physics and chemistry identification according to Chinese pharmacopoeia [1]. A voucher specimen (LICH-2012-SC) was deposited in the Institute of Traditional Chinese Medicine and Natural Products, Jinan University (China).

### Extraction and isolation

The dried rhizome of *L. chuanxiong* Hort. (18.0 kg) was cut into small pieces and extracted with 60 % ethanol (70 L × 2 h × 2). The concentrated extract (5.69 kg) was suspended in H_2_O (13 L) and successively partitioned with EtOAc and *n*-BuOH (each 10 L × 4). The EtOAc layer (480 g) was loaded onto a silica gel column (8 × 75 cm) and eluted with a cyclohexane–EtOAc gradient (100:0, 99:1, 98:2, 95:5, 90:10, 80:20, 70:30, 60:40, 0:100, *v/v*, each 35 L) to produce 13 fractions (Fr.1–Fr.13). Fr.2 (20.0 g) was loaded onto an ODS column (4 × 35 cm) and eluted with a MeOH–H_2_O gradient (40:60, 50:50, 60:40, 70:30, 80:20, 100:0, *v/v*, each 1 L) to give 5 fractions (Fr.2.1–Fr.2.5). Fr.2.2 (397 mg) was separated by semipreparative HPLC (45 % MeOH–H_2_O, 3.5 mL/min) to afford compounds **7** (t_R_ = 30.2 min; 32.4 mg) and **8** (t_R_ = 26.4 min; 25.9 mg). Fr.8 (17.0 g) was applied to an ODS column (4 × 44 cm) and eluted with a MeOH–H_2_O gradient (10:90, 30:70, 40:60, 50:50, 60:40, 70:30, 80:20, 90:10, 100:0, *v/v*, each 2 L) to give 17 subfractions (Fr.8.1–8.17). Fr.8.6 (1.09 g) was separated on a Sephadex LH-20 column (2 × 90 cm) by elution with a MeOH–H_2_O gradient (50:50, 60:40, 100:0, *v/v*, each 0.5 L) to yield 8 subfractions. Subfraction Fr.8.6.6 was purified by semipreparative HPLC (50 % MeOH-H_2_O, 4 mL/min) to yield compounds **9** (t_R_ = 18.6 min; 9.8 mg) and **10** (t_R_ = 22.1 min; 17.3 mg). Fr.8.17 (0.98 g) was separated on a Sephadex LH-20 column (3 × 120 cm) using MeOH to yield 6 subfractions. Subfraction Fr.8.17.3 (899.0 mg) was further purified by preparative HPLC (87 % MeOH–H_2_O, 8 mL/min) to yield compounds **1** (t_R_ = 156 min; 36.0 mg), **2** (t_R_ = 147 min; 10.7 mg), **3** (t_R_ = 173 min; 19.1 mg), **4** (t_R_ = 164 min; 4.0 mg), **5** (t_R_ = 114 min; 137.0 mg), and **6** (t_R_ = 107 min; 33.6 mg). Chiral analyses of compounds **1**–**6** (95 % MeOH–H_2_O, 0.7 mL/min) and **7**–**8** (75 % MeOH–H_2_O, 0.5 mL/min) were conducted on a Lux TF Cellulose-2 column. For compound **1**, chiral separation was performed on the same chiral column (95 % MeOH–H_2_O, 0.7 mL/min) to yield (+)-**1** (t_R_ = 11.8 min; 1.0 mg) and (−)-**1** (t_R_ = 16.2 min; 1.0 mg).

## Results

**Fig. 1:**
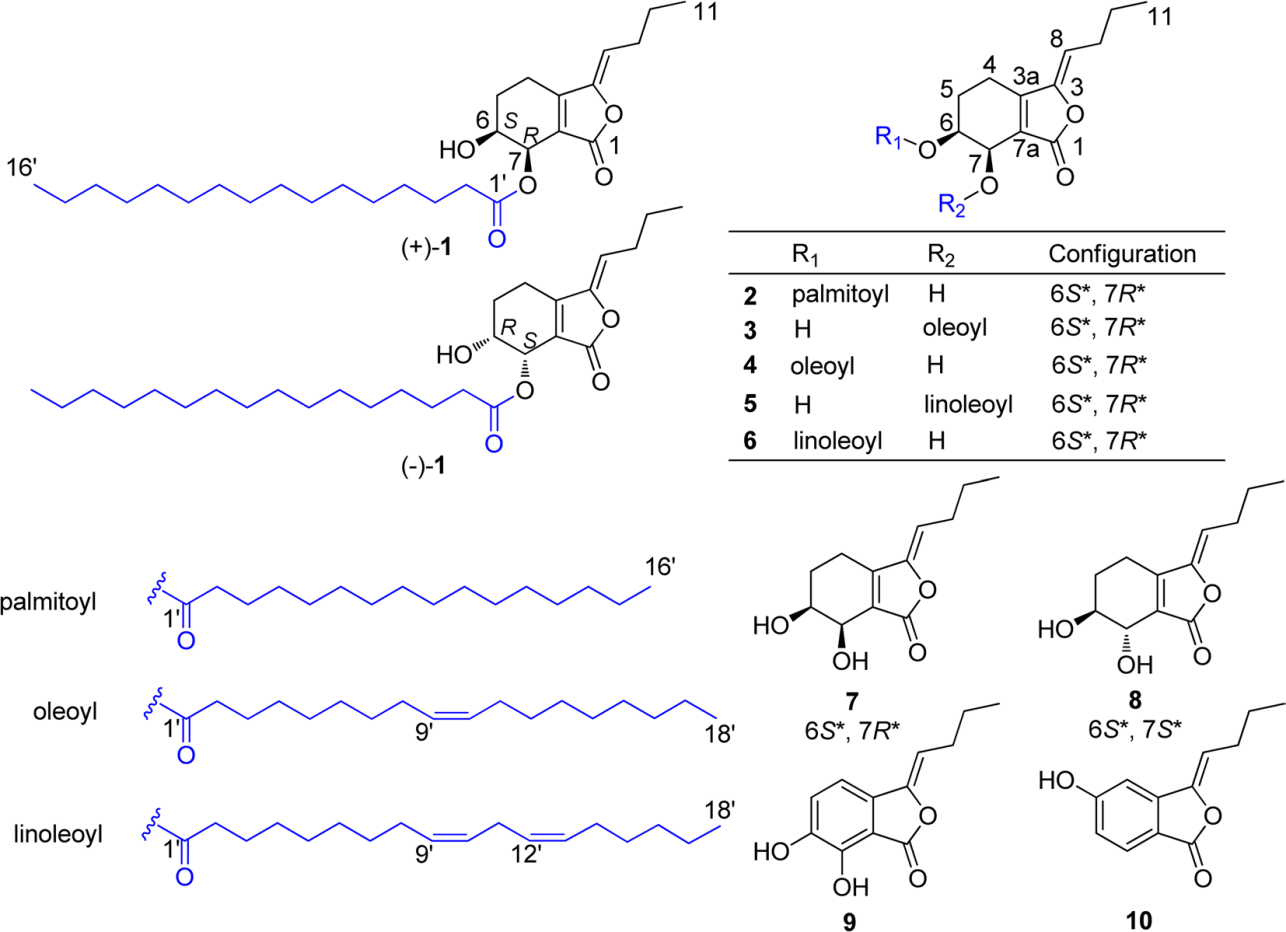
Structures of compounds **1**–**10**

Six novel phthalide derivatives, (+)/(−)-chuanxiongins A–F (**1**–**6**), a new natural product (**9**), and three known phthalides (**7**, **8**, and **10**) were isolated from the 60 % ethanol extract of *Chuanxiong* (Fig. 1). Compounds **1**–**8** were obtained as pairs of enantiomers. Compound **1** was successfully resolved by chiral HPLC to afford (+)-**1** and (−)-**1**. Their structures and absolute configuration were elucidated on the basis of spectroscopic data, circular dichroism (CD) analyses, and the modified Mosher’s method.

### Physicochemical data of new compounds

Chuanxiongin A (**1**). Colorless oil; UV (MeOH) *λ*_max_ (log *ε*) 204 (4.03), 275 (4.09) nm; IR (KBr) *ν*_max_ 3446, 2955, 2930, 2858, 1742, 1458 cm^−1^; ^1^H NMR (CDCl_3_, 300 MHz) and ^13^C NMR (CDCl_3_, 75 MHz), see Tables 1 and 2; HR-ESI-MS *m/z* 463.3421 [M + H]^+^ (calcd for C_28_H_47_O_5_, 463.3423).

**Table 1:** 1H and 13C NMR data for the phthalide nucleus of **1**–**8** (CDCl_3_, δ in ppm, J in Hz)*

Position	**1**/**3**/**5**		**2**/**4**/**6**		**7**		**8**	
	*δ*_C_	*δ*_H_	*δ*_C_	*δ*_H_	*δ*_C_	*δ*_H_	*δ*_C_	*δ*_H_
1	167.7		168.9		169.6		169.4	
3	148.2		148.3		148.3		148.1	
3a	155.2		152.9		153.7		153.3	
4	17.8	2.48 (dt, 18.5, 5.4) 2.62 (dt, 18.5, 6.9)	18.3	2.51	18.9	2.41 2.63 (dt, 18.5, 5.8)	19.1	2.51
5	25.8	1.99	23.1	2.00 2.10	25.5	1.82 2.08	26.5	1.88 2.06
6	68.5	4.08 (dt, 6.0, 3.9)	72.3	5.10	67.9	4.00	71.7	3.93
7	67.5	5.53 (d, 3.9)	63.4	4.56 (d, 3.9)	63.0	4.59 (d, 3.0)	67.6	4.44 (d, 5.5)
7a	122.3		125.6		125.7		125.9	
8	114.3	5.31 (t, 7.9)	114.5	5.31 (t, 7.9)	114.7	5.30 (t, 7.9)	114.6	5.28 (t, 7.9)
9	28.2	2.36	28.3	2.37	28.2	2.34	28.2	2.33
10	22.4	1.50	22.4	1.51	22.4	1.49	22.4	1.47
11	13.9	0.95 (t, 7.4)	13.9	0.96 (t, 7.4)	13.9	0.94 (t, 7.4)	13.9	0.93 (t, 7.3)

* Indiscernible signals owing to overlapping or having complex multiplicity are reported without designating multiplicity

**Table 2:** 1H and 13C NMR data for the fatty acid moieties of **1**–**6** (CDCl_3_, δ in ppm, J in Hz)*

Position	Palmitoyl for **1** and **2**		Oleoyl for **3** and **4**		Linoleoyl for **5** and **6**	
	*δ*_C_	*δ*_H_	*δ*_C_	*δ*_H_	*δ*_C_	*δ*_H_
1′	174.0 for **1** 173.3 for **2**		174.0 for **3** 173.3 for **4**		174.0 for **5** 173.3 for **6**	
2′	34.4	2.32 for **1** 2.29 for **2**	34.4	2.33 for **3** 2.30 for **4** 1.62 for **3** 1.60 for **4**	34.4	2.33 for **5** 2.29 for **6**
3′	25.0	1.62 for **1** 1.59 for **2**	25.0		25.0	1.63 for **5** 1.59 for **6**
4′	29.2–29.8	1.22–1.32	29.2–29.9	1.24–1.37	29.2–29.7	1.24–1.37
5′	29.2–29.8	1.22–1.32	29.2–29.9	1.24–1.37	29.2–29.7	1.24–1.37
6′	29.2–29.8	1.22–1.32	29.2–29.9	1.24–1.37	29.2–29.7	1.24–1.37
7′	29.2–29.8	1.22–1.32	29.2–29.9	1.24–1.37	29.2–29.7	1.24–1.37
8′	29.2–29.8	1.22–1.32	27.4	2.01	27.3	2.04
9′	29.2–29.8	1.22–1.32	129.9	5.35	130.2	5.36
10′	29.2–29.8	1.22–1.32	130.1	5.35	128.2	5.33
11′	29.2–29.8	1.22–1.32	27.3	2.01	25.8	2.76 (t, 6.0)
12′	29.2–29.8	1.22–1.32	29.2–29.9	1.24–1.37	128.1	5.33
13′	29.2–29.8	1.22–1.32	29.2–29.9	1.24–1.37	130.4	5.36
14′	32.1	1.24	29.2–29.9	1.24–1.37	27.3	2.04
15′	22.8	1.27	29.2–29.9	1.24–1.37	29.2–29.7	1.24–1.37
16′	14.3	0.87 (t, 6.7)	32.0	1.25	31.7	1.28
17′			22.8	1.29	22.7	1.29
18′			14.2	0.87 (t, 6.7)	14.2	0.88 (t, 6.8)

* Indiscernible signals owing to overlapping or having complex multiplicity are reported without designating multiplicity

#### (+)-Chuanxiongin A [(+)-**1**]

[*α*]^17^_D_ + 35.5 (*c* 0.40, MeOH); CD (MeOH) *λ*_max_ (Δ*ε*) 249.2 (-0.12), 220.6 (+0.72) nm.

#### (−)-Chuanxiongin A [(−)-**1**]

[*α*]^17^_D_-31.6 (*c* 0.35, MeOH); CD (MeOH) *λ*_max_ (Δ*ε*) 251.3 (–0.13), 221.5 (+0.63) nm.

#### (S)-MTPA ester of (−)**-1** [(−)-**1-a**]

^1^H NMR (C_5_D_5_N, 400 MHz): *δ*_H_ 2.6001 (2H, m, H-4), 2.2082 (2H, overlapped, H-5), 5.7335 (1H, m, H-6), 6.2526 (1H, d, *J* = 5.5 Hz, H-7).

#### (R)-MTPA ester of (−)**-1** [(−)-**1-b**]

^1^H NMR (C_5_D_5_N, 400 MHz): *δ*_H_ 2.5415 (1H, overlapped, H-4), 2.3910 (1H, dt, *J* = 18.5, 6.1 Hz, H-4), 2.1479 (2H, overlapped, H-5), 5.7616 (1H, m, H-6), 6.3017 (1H, d, *J* = 5.1 Hz, H-7).

#### Chuanxiongin B (**2**)

Colorless oil; UV (MeOH) *λ*_max_ (log *ε*) 205 (4.00), 275 (3.96) nm; IR (KBr) *ν*_max_ 3446, 2957, 2925, 2854, 1771, 1741, 1457 cm^−1^; ^1^H NMR (CDCl_3_, 300 MHz) and ^13^C NMR (CDCl_3_, 75 MHz), see Tables 1 and 2; HR-ESI-MS *m/z* 463.3435 [M + H]^+^ (calcd for C_28_H_47_O_5_, 463.3423).

#### Chuanxiongin C (**3**)

Colorless oil; UV (MeOH) *λ*_max_ (log *ε*) 205 (3.83), 275 (3.81) nm; IR (KBr) *ν*_max_ 3422, 2956, 2926, 2854, 1774, 1741, 1459, 724 cm^−1^; ^1^H NMR (CDCl_3_, 300 MHz) and ^13^C NMR (CDCl_3_, 75 MHz), see Tables 1 and 2; HR-ESI-MS *m/z* 511.3394 [M + Na]^+^ (calcd for C_30_H_48_O_5_Na, 511.3399).

#### Chuanxiongin D (**4**)

Colorless oil; UV (MeOH) *λ*_max_ (log *ε*) 204 (3.96), 275 (4.00) nm; IR (KBr) *ν*_max_ 2956, 2926, 2855, 1771, 1742, 1457 cm^−1^; ^1^H NMR (CDCl_3_, 400 MHz) and ^13^C NMR (CDCl_3_, 100 MHz), see Tables 1 and 2; HR-ESI-MS *m/z* 489.3579 [M + H]^+^ (calcd for C_30_H_49_O_5_, 489.3580).

#### Chuanxiongin E (**5**)

Colorless oil; UV (MeOH) *λ*_max_ (log *ε*) 205 (3.91), 274 (3.45) nm; IR (KBr) *ν*_max_ 3421, 2956, 2929, 2857, 1732, 1716, 1457 cm^−1^; ^1^H NMR (CDCl_3_, 300 MHz) and ^13^C NMR (CDCl_3_, 75 MHz), see Tables 1 and 2; HR-ESI-MS *m/z* 509.3250 [M + Na]^+^ (calcd for C_30_H_46_O_5_Na, 509.3243).

#### Chuanxiongin F (**6**)

Colorless oil; UV (MeOH) *λ*_max_ (log *ε*) 205 (4.04), 273 (3.53) nm; IR (KBr) *ν*_max_ 3446, 2956, 2927, 2856, 1733, 1718, 1457 cm^−1^; ^1^H NMR (CDCl_3_, 300 MHz) and ^13^C NMR (CDCl_3_, 75 MHz), see Tables 1 and 2; HR-ESI-MS *m/z* 509.3243 [M + Na]^+^ (calcd for C_30_H_46_O_5_Na, 509.3243).

## Discussion

### Structure elucidation of compounds

**Fig. 2:**
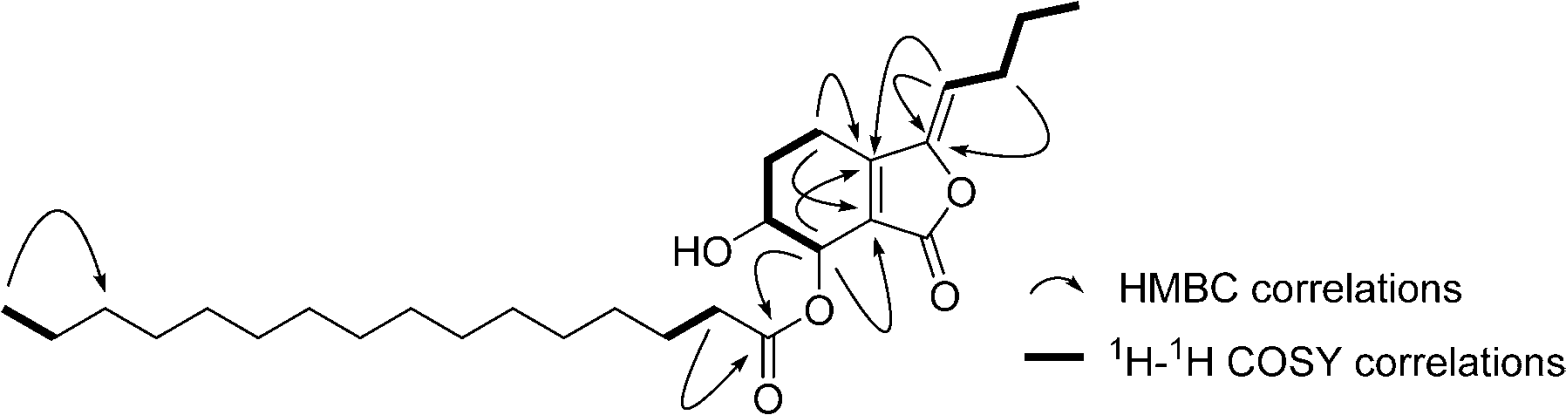
Key HMBC and ^1^H-^1^H COSY correlations of 1

Chuanxiongin A (**1**), obtained as a colorless oil, was assigned the molecular formula C_28_H_46_O_5_ according to its HR-ESI-MS at *m/z* 463.3421 [M + H]^+^ (calcd for 463.3423). The ^1^H-NMR spectral data (Tables 1 , 2) of **1** were similar to those of senkyunolide H (**7**) [5], expect for the appearance of typical fatty acid signals (*δ*_H_ 1.22–2.32 and *δ*_C_ 22.8–34.4). Indeed, the presence of the senkyunolide H moiety was further confirmed by detailed analyses in ^1^H-^1^H COSY, HSQC, and HMBC experiments (Fig. 2). On the basis of the mass spectral information, the fatty acid moiety was deduced as palmitic acid. The above spectral data suggested that **1** might be the product of **7** acylated at 6-OH or 7-OH with a long-chain fatty acyl moiety. Furthermore, from the significant downfield shift [*δ*_H_ 5.53 (d, *J* = 3.9 Hz, H-7)] in comparison with **7**, the palmitoyl group was assigned to 7-OH via an ester bond. This was supported by the key HMBC correlation from H-7 to the ester carbonyl at *δ*_C_ 174.0 of the palmitoyl group. The relative stereochemistry of **1** was found to be the same as **7** (*cis*-oriented) owing to the small *J*_6,7_ value of 3.9 Hz, as well as the carbon chemical shifts of C-6 and C-7 [16]. Thus, the structure of **1** was elucidated as (4R*,5S*,*Z*)-1-butylidene-5-hydroxy-3-oxo-1,3,4,5,6,7-hexahydroisobenzofuran-4-yl palmitate, and designated chuanxiongin A.

**Fig. 3:**
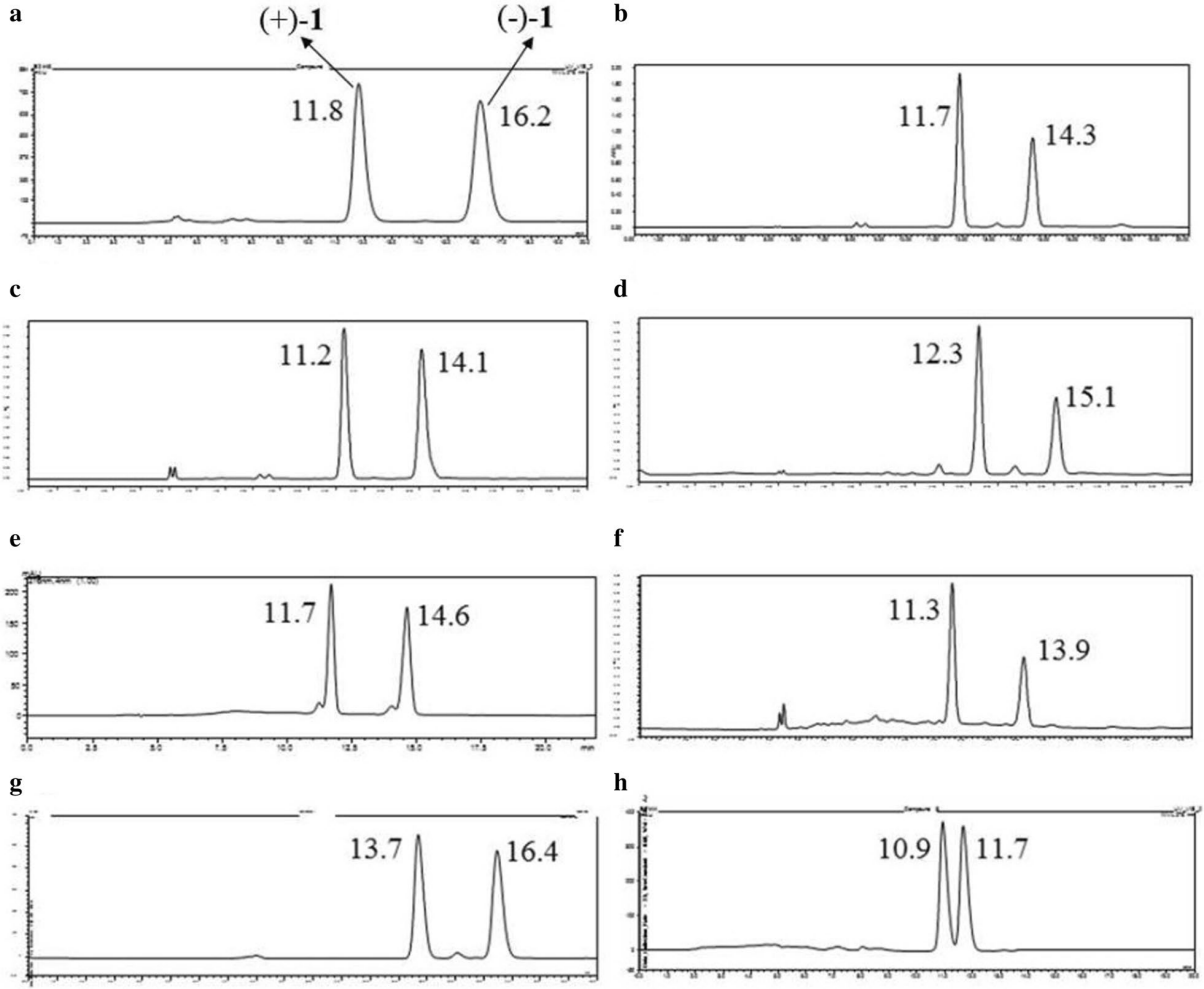
Chiral HPLC analytical chromatograms of compounds **1**–**8**. The retention times (min) for the two peaks of compounds **1**–**6** (95 % MeOH-H_2_O, 0.7 mL/min; 276 nm) and **7**–**8** (75 % MeOH-H_2_O, 0.5 mL/min; 276 nm) are marked on each chromatogram. The letters **a–h** successively stand for the profiles of **1**–**8**

**Fig. 4:**
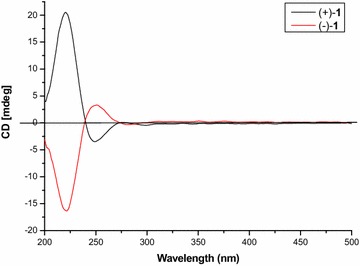
CD spectra of (+)-**1** and (−)-**1**

**Fig. 5:**
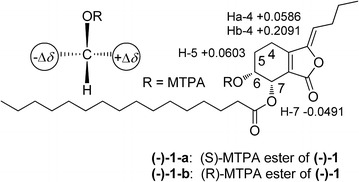
The Δδ values of the (S)- and (R)-MTPA esters ((−)-**1-a** and (−)-**1-b**) indicating the R configuration of C-6 in (−)-**1**

The optical rotation value of **1** was almost zero, indicating a racemic nature, which was in accordance with the appearance of two peaks in the chiral HPLC analysis (Fig. 3). The chiral resolution of **1** by chiral HPLC afforded a pair of anticipated enantiomers, (+)-**1** and (−)-**1**, which were opposite in terms of their optical values and CD curves (Fig. 4). The absolute configuration of the enantiomer (−)-**1** was established by the modified Mosher’s method [17]. A solution of (−)**-1** (0.5 mg) in C_5_D_5_N (0.5 mL) was treated with (*R*)-α-methoxy-α-trifluoromethyl-α-phenylacetyl chloride ((*R*)-MTPA chloride) (10 μL) under an atmosphere of nitrogen in an NMR tube. The mixture was stirred at room temperature for 4 h to obtain the (*S*)-MTPA ester [(−)-**1-a**]. The same procedure was used to prepare the (*R*)-MTPA ester [(−)-**1-b**] with (*S*)-MTPA chloride. The Δ*δ* values of the (*S*)- and (*R*)-MTPA esters [(−)-**1-a** and (−)-**1-b**, respectively] indicated the *R* configuration for C-6 (Fig. 5). Therefore, the absolute configurations of (+)-**1** and (−)-**1** were respectively established as *6S*, *7R* and *6R*, *7S*.

Chuanxiongin B (**2**) had the same molecular formula of C_28_H_46_O_5_ as **1**, obtained from its HR-ESI-MS at *m/z* 463.3435 [M + H]^+^, and exhibited similar physical and NMR data (Tables 1 and 2) to those of **1**, except for the large upfield shift of H-7 and downfield shift of H-6, indicating the position of esterification at 6-OH instead of 7-OH. This deduction was further confirmed by the key HMBC correlation from H-6 to the ester carbonyl at *δ*_C_ 173.3. Therefore, the structure of **2** was deduced as senkyunolide H 6-palmitate, with the systematic name of (4R*,5S*,*Z*)-1-butylidene-4-hydroxy-3-oxo-1,3,4,5,6,7-hexahydroisobenzofuran-5-yl palmitate. It was designated with the common name chuanxiongin B.

Chuanxiongin C (**3**), a colorless oil, was assigned with the molecular formula C_30_H_48_O_5_ by HR-ESI-MS (*m/z* 511.3394 [M + Na]^+^). The ^1^H- and ^13^C-NMR data of the phthalide moiety in **3** were essentially identical to those of **1** (Table 1). The only difference was an oleoyl group in **3** instead of a palmitoyl group in **1**. The presence of the oleoyl group was further confirmed on the basis of the mass and NMR spectral data [18]. Accordingly, compound **3** was determined as (4R*,5S*,*Z*)-1-butylidene-5-hydroxy-3-oxo-1,3,4,5,6,7-hexahydroisobenzofuran-4-yl oleate, and designated chuanxiongin C.

Chuanxiongin D (**4**) was determined to have the same molecular formula as **3** on the basis of the HR-ESI-MS (*m/z* 489.3579 [M + H]^+^) and NMR spectra. The ^1^H- and ^13^C-NMR spectral data of **4** showed the signals for oleoyl and senkyunolide H moieties. In the HMBC spectrum, the proton H-6 showed a long-range correlation with the ester carbonyl, indicating that the fatty acid chain was linked to 6-OH. Thus, **4** was determined as (4R*,5S*,*Z*)-1-butylidene-4-hydroxy-3-oxo-1,3,4,5,6,7-hexahydroisobenzofuran-5-yl oleate, and designated chuanxiongin D.

On the basis of the HR-ESI-MS (*m/z* 509.3250 [M + Na]^+^ for **5**; 509.3243 [M + Na]^+^ for **6**) and NMR spectra, the molecular formula of chuanxiongin E (**5**) and chuanxiongin F (**6**) was determined to be C_30_H_46_O_5_. According to the NMR evidence, **5** and **6** were also fatty acid-acylated derivatives of senkyunolide H (**7**). Moreover, the acylating fatty acid moiety was deduced to be a linoleoyl group from the mass spectral data, as well as a comparison of the ^13^C NMR data with previous literature [18]. Similarly, the HMBC correlations and considerable acylation shifts implied that in the cases of **5** and **6**, a linoleoyl group was attached at the 7-OH and 6-OH positions, respectively. Thus, **5** and **6** were determined as (4R*,5S*,*Z*)-1-butylidene-5-hydroxy-3-oxo-1,3,4,5,6,7-hexahydroisobenzofuran-4-yl linoleate (**5**) and (4R*,5S*,*Z*)-1-butylidene-4-hydroxy-3-oxo-1,3,4,5,6,7-hexahydroisobenzofuran-5-yl linoleate (**6**), and designated chuanxiongin E (**5**) and chuanxiongin F (**6**), respectively.

Compounds **7** and **8** were identified as senkyunolide H (**7**) [5] and senkyunolide I (**8**) [5] by comparisons of their physiochemical properties and spectral data with those reported in the literature.

Similarly, the optical rotation values of compounds **2**–**8** were almost zero, suggesting that they were also present as pairs of enantiomers, as further confirmed by the observations of two separated peaks in the chiral chromatographic analysis experiment (Fig. 3).

Compounds **9** and **10** were elucidated as 6-hydroxy-senkyunolide B (**9**) [19, 20] and senkyunolide C (**10**) [21] based on comparisons with published spectroscopic data.

Chuanxiongins A–F (**1**–**6**) comprise a new subtype of phthalides and represent a new class of chemical constituents in *Chuanxiong*. The discovery of them warranted further research on its mechanism of action and use for quality control.

## Conclusions

Six novel phthalide derivatives (**1**–**6**) isolated from *Chuanxiong* were phthalide fatty acid esters that were structurally analogous and characterized by fatty acid acylation at 6-OH or 7-OH.
